# A novel TIRADS of US classification

**DOI:** 10.1186/s12938-018-0507-3

**Published:** 2018-06-18

**Authors:** Yan Zhuang, Cheng Li, Zhan Hua, Ke Chen, Jiang Li Lin

**Affiliations:** 1 Department of Biomedical Engineering, Sichuan University College of Materials Science and Engineering, Chengdu, 610065 Sichuan China; 2 China-Japan Friendship Hospital, Beijing, 100029 China

**Keywords:** Thyroid nodules, TIRADS ultrasound, Feature extraction, Classification

## Abstract

**Background:**

Thyroid imaging reporting and data system (TIRADS) is the assessment of a risk stratification of thyroid nodules, usually using a score. However, there is no consensus as to the version of TIRADS for reporting the results of thyroid ultrasound in clinic. The objective of this study is to develop a practical TIRADS with which to categorize thyroid nodules and stratify their malignant risk.

**Methods:**

A TIRADS scoring system was developed to provide more decision levels than standard scoring through the selection of the ultrasound features which include the calcification shape, margins, taller-than-wide, internal echo, blood flow quantization of features, setting of the weight, and calculation of the score. Ultimately, the accuracy of our TIRADS was evaluated by comparing with the results of current vision of TIRADS and thyroid radiologist in 153 patients who had US-guided fine-needle aspiration biopsy.

**Results:**

Classification results showed that the total accuracy reached 97% (100% of malignant and 95% of the benign) in 153 cases (benign:78, malignant:75). The percentages of malignancy is defined in our TIRADS were as follows: TIRADS 2 (0% malignancy), TIRADS 3 (3.6% malignancy), TIRADS 4 (17–75% malignancy), and TIRADS 5 (98% malignancy).

**Conclusions:**

We established a novel TIRADS to predict the malignancy risk of the thyroid nodules based on six categories US features by a scoring system, which included a standardized vocabulary and score and a quantified risk assessment. The results showed that objective quantitative classification of thyroid nodules by our TIRADS can be useful in guiding management decisions.

## Background

The prevalence of thyroid nodules in population is increasing around the world. In China, the morbidity of thyroid cancer grows gradually from year to year, especially in female patients [1–3]. Recently, the estimated incidence of thyroid nodules is nearly 19–67%, approximately 5–15% of these nodules are found to be malignant [4]. Thyroid ultrasound (US) is a key examination for the management of thyroid nodules. Thyroid US is easily accessible, noninvasive, cost-effective, and is a mandatory step in the diagnosis of thyroid nodules [5]. Thus, it is necessary to standardize terminology and create guidelines to categorize thyroid nodules according to their malignant potential for effective management [6].

Several different thyroid imaging reporting and data system (TIRADS) classifications and recommendations have been proposed [7–10]. In 2009, Horvath et al. [7] described 10 US patterns of thyroid nodules and divided these nodules into a 5-point TIRADS with malignancy risk. However, their system is difficult to apply because not all thyroid nodules have stereotypic appearances on US. Park et al. [8] provided an equation for predicting the probability of malignancy in thyroid nodules on the basis of 12 US features. The categorization may be difficult to apply in practice because it requires subjective judgment of doctors on suspicious features and complex calculations. Recently, Kwak et al. [9] used multivariate regression analysis and proposed a TIRADS score that refers to five risk features: micro-calcification, irregular shape, taller-than-wide, solidity, and hypoechogenicity. As the number of suspicious US features increased, risk of malignancy also increased. They developed a 5-grade scale with a score of 2 for benign lesions; 3 for no suspicious features; 4A, 4B, and 4C when there were one, two, and three or four suspicious features, respectively; and 5 when all the six risk factors were presented. This system is convenient for risk stratification, and simple to use. However, each US feature of this TIRADS is given the same weight, without consideration of the different probabilities of malignancy associated with each, and the determination of the feature performance in TIRADS depends on the doctors.

It is widely accepted that no single US feature has enough sensitivity and specificity to reliably indicate that thyroid nodules are benign or malignant, many US features of TIRADS have inter- and intra-observer variation, making difficult an accurate diagnosis based on TIRADS. Each US feature has different effects on the malignant evaluation of thyroid nodules but none covers US features weights reasonably in previous TIRADS. The certainty of malignancy increases with the number of features rather than an available comprehensive threshold from the US features.

In this study, we established a novel TIRADS that provided many potential decision levels by distinguishing weights among the features of six categories, quantifying each malignant risk indicators through a TIRADS scoring system. Ultimately, the goal was to obtain an objective and comprehensive evaluation of each thyroid nodules based on our TIRADS.

## Methods

### Features

This paper put forward 6 category features of TIRADS through studying with clinical experts, as shown in Table 1. The composition feature includes the performance of solid, cystic and mixed [11]. The feature of margin is evaluated by ill-defined and microlobulated. The shape of the tumor is quantified with degree of irregularity. The feature of calcification was divided into micro-calcification, macro-calcification and no-calcification. The distributions of blood flow are characterized as central type, peripheral type, messy type, focal thyroid inferno (Doppler flow covering the entire nodule whereas little or no flow within the surrounding parenchyma [12]) and no blood flow signals. These features corresponding to their manifestations play an important role in predicting benign and malignant thyroid nodules.

**Table 1:** TIRADS classification features of thyroid ultrasound

Feature category	US features				
Composition	Solid		Cystic	Mixed	
Margin	Ill-defined			Microlobulated	
Shape	Irregular				
Calcification	Micro-calcification		Macro-calcification	No-calcification	
Taller than wide	<= 1 (wider than tall)			> 1 (taller than wide)	
Blood flow	Central	Peripheral	Messy	Focal thyroid inferno	No

### Features quantification

#### Composition

**Fig. 1:**
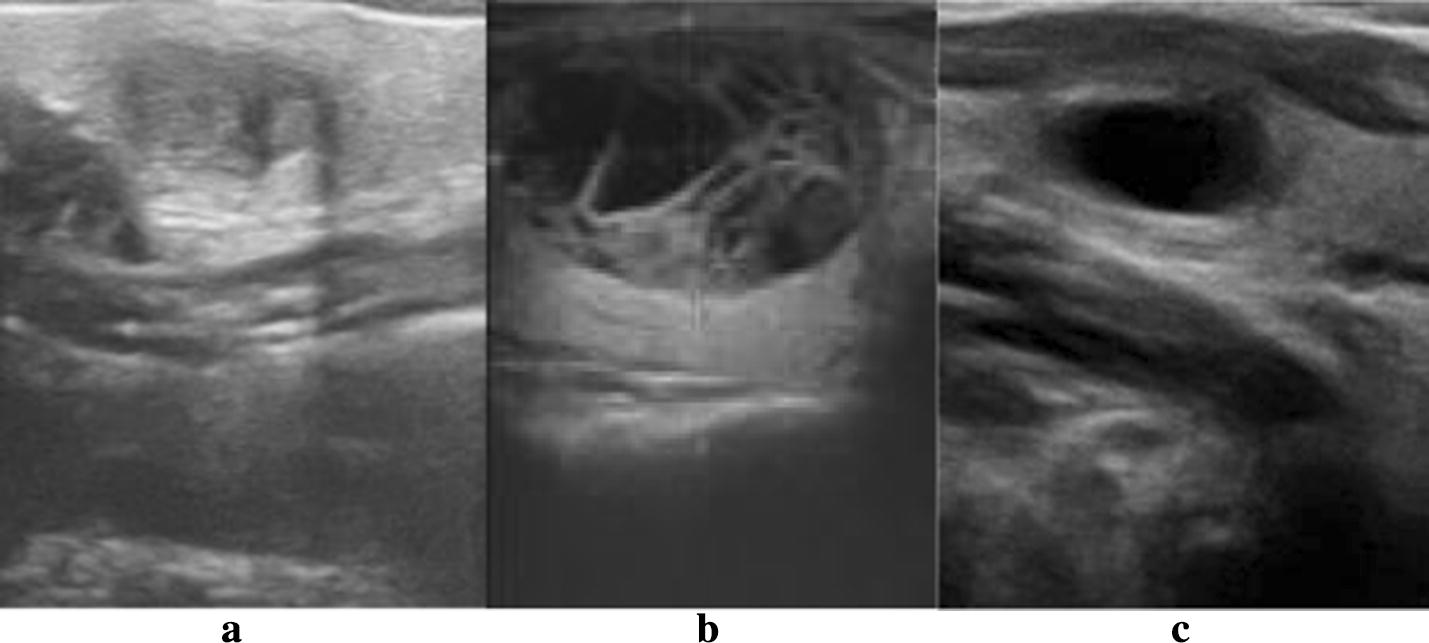
Feature of internal composition for thyroid nodules. Three types of internal components of thyroid nodules, they are **a** solid, **b** mixed and **c** cystic

It is widely accepted that the internal composition of benign tumor is mainly cystic, malignant tumor is mainly solid. Predominantly solid composition of mixed tumor is commonly malignant. Three types of internal components of thyroid nodules, as shown in Fig. 1.

Quantify the composition characteristics of tumor based on the gray-level histogram. First, get gray histogram of the tumor region which number of gray pixel is not zero, and count the top 10 and 5% of the pixels number of the gray level distribution: *N*_1_, *N*_2_ respectively, and the number *N*_0_ of the pixel gray level 0, then the top 50% and the remaining 50% of the number of pixels of the gray level distribution respectively, the gray variance *V*_1_, *V*_2_ and pixels number *N* of the nodule region. The cystic rate is defined as *CysR*, as shown in Formula (1), and quantitative formula of the composition Com is shown in (2).

1 $$CysR = {{\left( {N_{1} + N_{2} } \right)} \mathord{\left/ {\vphantom {{\left( {N_{1} + N_{2} } \right)} N}} \right. \kern-0pt} N}$$

2 $$Com = \left\{ {\begin{array}{*{20}l} {Solid,\;CysR \le 0.02,\;V_{1} \le V_{2} } \\ {Cystic,\;CysR \ge 0.3,\;N_{1} > N_{2} ,\;N_{0} > 0} \\ {Mixed,\;Other} \\ \end{array} } \right.$$

#### Shape

**Fig. 2:**
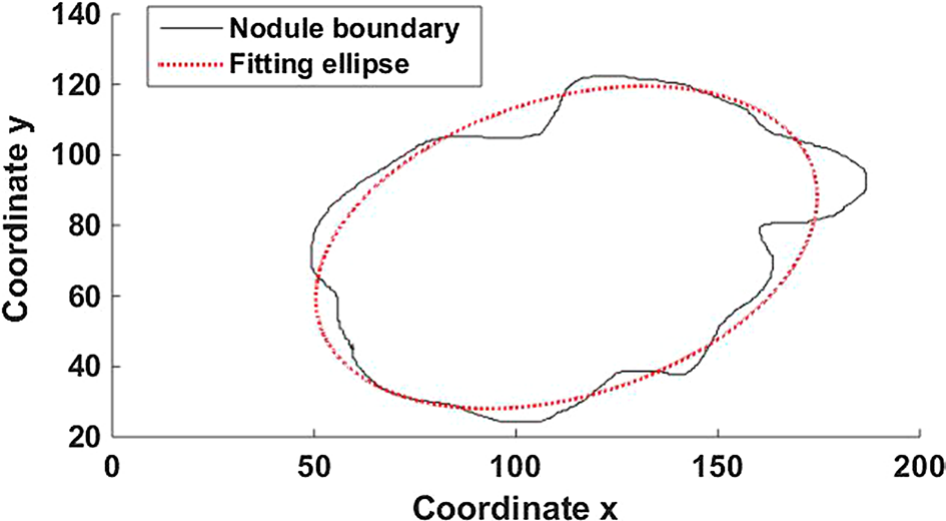
The boundary fitted curve of thyroid nodule. The thyroid nodule boundary and boundary fitted curve, where the black solid line is the nodule boundary and the red dashed line is the elliptic curve fitted based on this boundary

*Concavity* and *Compactness* are extracted automatically to quantify the shape of the irregularities in this paper. As the value of these parameters increases, the more irregular of thyroid nodules, and the increasing risk of malignancy will be. First, fit the quadratic curve of thyroid nodule boundaries by the least square method, as shown in Fig. 2.

For the feature of shape, two shape parameters of *Concavity* and *Compactness* are extracted to quantify degree of the irregularities in this paper. The more irregular the thyroid nodules are, the more risk of malignancy there will be, and the greater the value of these parameters will be.

In order to obtain this parameter of Concavity, the quadratic curve was used to fit the boundary of thyroid nodules with the least square method firstly, as shown in Fig. 2.

So the fitted curve divides the nodule area into three parts, the concave part, which is beyond the boundary and within the curve, the overlapping part between the nodular area and the curve, and the convex which is inside the boundaries and outside the curve. *Concavity* is defined as the ratio of the sum area of concave and convex to the common area, as shown in Eq. (3).

3 $${\text{Concavity}} = \frac{{{\text{S}}_{\text{o}} + {\text{S}}_{\text{i}} }}{{{\text{S}}_{\text{c}} }},$$

wherein *S*_*o*_, *S*_*i*_ are the area of the convex part and the area of the concave part, respectively, *S*_*c*_ is the area of the nodular area overlapping with the curve.

Another parameter Compactness, is defined as the ratio of the square of the perimeter and area of thyroid nodule multiplied by 4π, as shown in Formula (4).

4 $${\text{Compactness}} = \frac{{{\text{L}}^{2} }}{{4\pi \times {\text{Area}}}}$$

wherein *L* is the perimeter, *Area* is the area of the nodule.

#### Margin

**Fig. 3:**
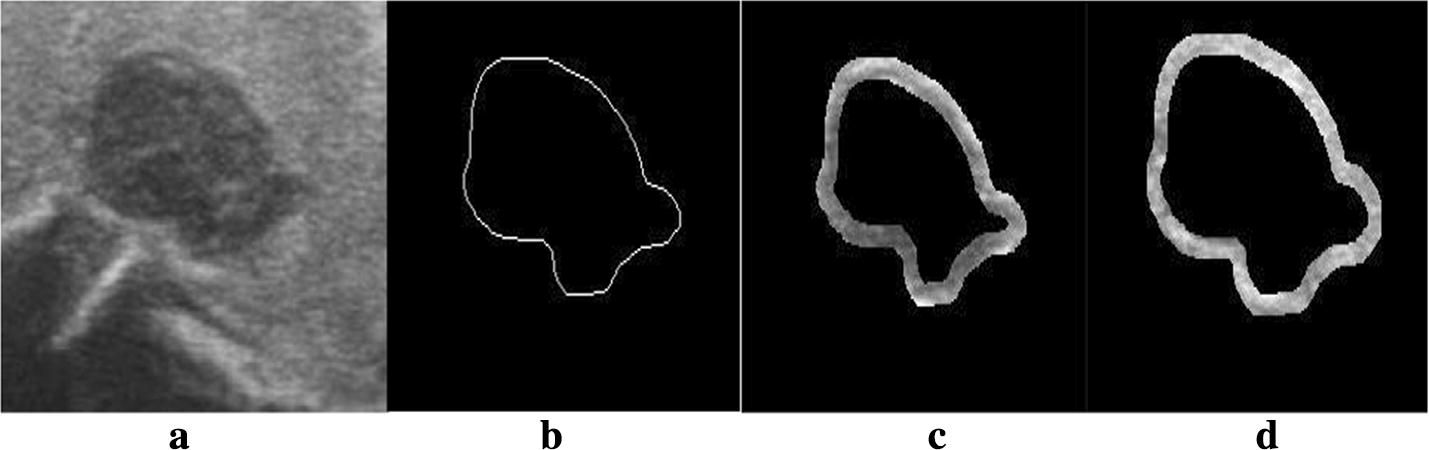
Feature of margin for thyroid nodules. The characteristics of the thyroid margin, **a** is the original image of thyroid nodule, **b** is the boundary of the nodule, and **c**, **d** are the internal and external bands of the boundary after morphological operation, respectively

The quantification of the margin is mainly based on the gray scale inside and outside the nodule boundary. A 10-pixel disk structure was used to obtain the band-shaped region in the binary images of the tumor region for erosion and dilation operations, as shown in Fig. 3.

Given that the number of pixels in the inner and outer band are represented by *n*_1_ and *n*_2_, and the mean gray values *u*_1_ and *u*_2_, respectively, the statistical difference of the gray scales between the inner and outer regions of the nodules adjacent to the border is measured by the inter-class variance [13], as shown in Formula (5).

5 $$InterVar = \frac{{n_{1} (u_{1} - u)^{2} + n_{2} (u_{2} - u)^{2} }}{{(n_{1} + n_{2} )}},\quad u = \frac{{n_{1} u_{1} + n_{2} u_{2} }}{{n_{1} + n_{2} }}$$

Next, normalized to get the average gray scale difference (mean separability),

6 $$MeanSep = \frac{InterVar}{TotalVar}$$

wherein *TotalVar* represents the variance of the gray levels of all the pixels in the banded region inside and outside the boundary.

#### Calcification

**Fig. 4:**
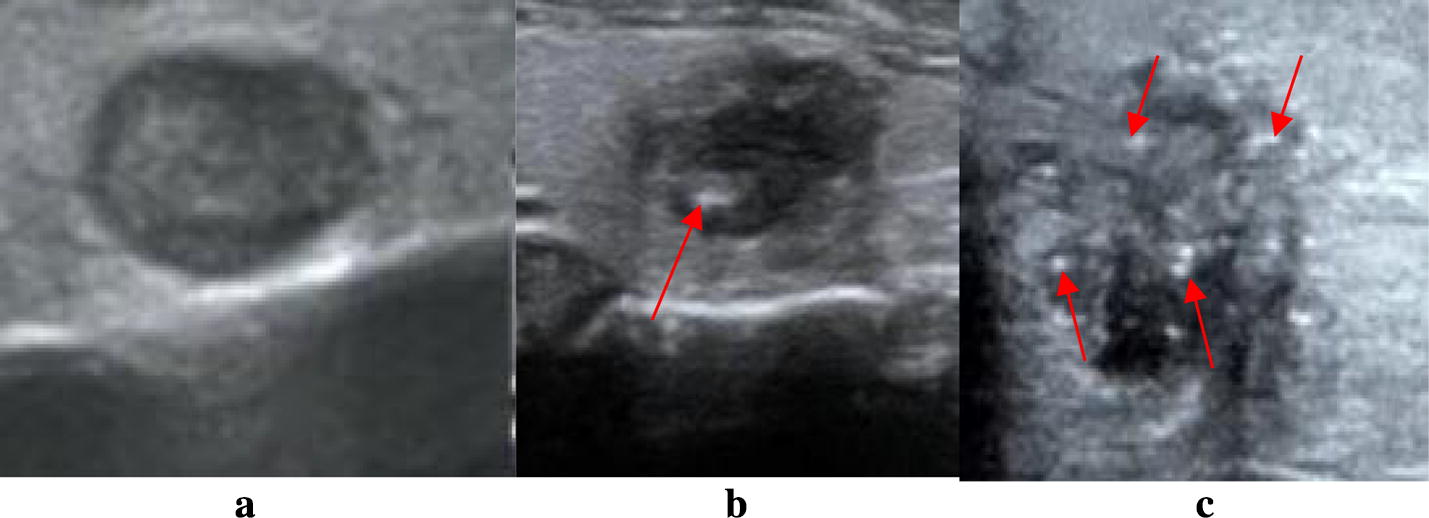
Feature of calcification for thyroid nodules. Three types of calcification features of thyroid nodules, they are no-calcification, macro-calcification and micro-calcification, and the calcification area is shown by the red arrow in **b**, **c**. **a** No-calcification, **b** macro-calcification, **c** micro-calcification

The features of calcification are mainly manifested as: macro-calcification, no-calcification and micro-calcification [14], as shown by the red arrows in Fig. 4. Micro-calcification is recognized as a strong indicator of malignant, and the macro-calcification and no-calcification also have the potential for malignancy. Deep learning algorithm [15] is used to quantify calcification into these three categories, which is not detailed here.

#### Taller than wide

**Fig. 5:**
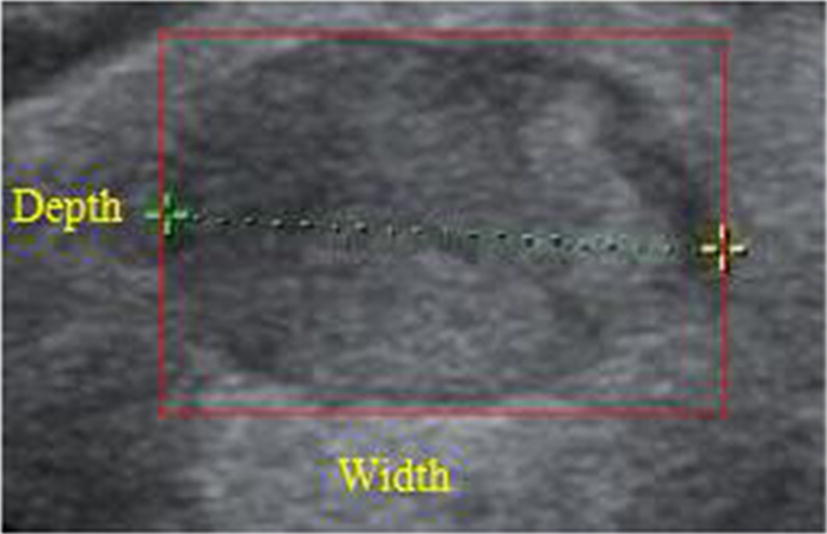
Feature of Taller than wide for thyroid nodules. The red rectangle in the figure is the minimum bounding rectangle of the thyroid nodule boundary, where depth and width are the length and width of the rectangle respectively

Taller than wide is another momentous feature of shape, called aspect ratio (AR), defined as the ratio of depth to width, as shown in Fig. 5. It reflects the growth pattern of tumor to a certain degree. If the greater *AR* is, the higher risk of malignancy.

#### Blood flow

**Fig. 6:**
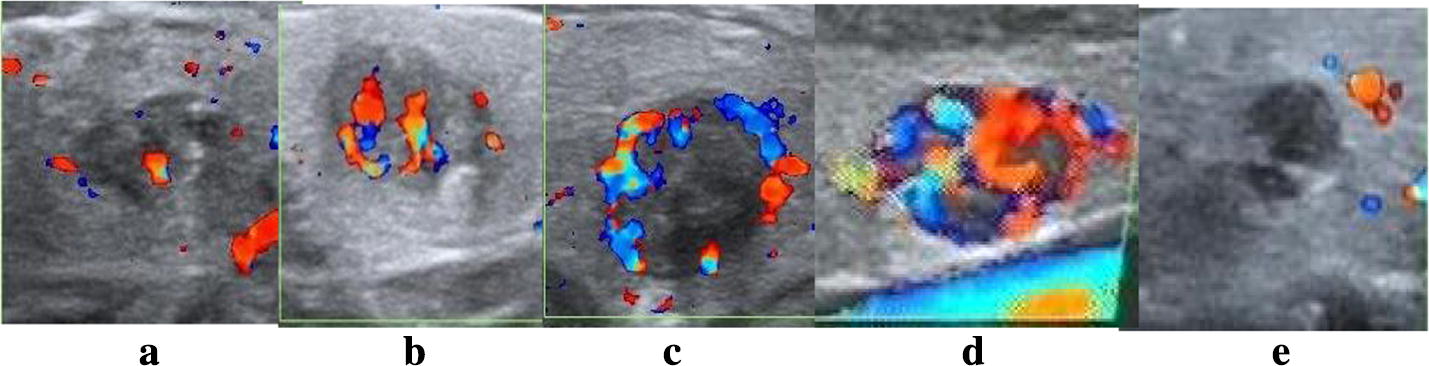
Thyroid nodules blood flow distribution. The distribution pattern of blood flow was quantified as: central type, peripheral type, focal thyroid inferno, messy type and no blood flow, as shown in figure. **a** Messy, **b** central type, **c** peripheral type, **d** focal thyroid inferno, **e** no vascularity

However, many other previous TIRADS studies did not contain the feature of blood flow. We believe that using color Doppler imaging is crucial to the improvement of the diagnostic accuracy of benign and malignant thyroid nodules, especially in the distribution of blood flow [16–19]. Generally, the blood flow distribution of central type is considered as one of the significant malignant features, and the distribution of focal thyroid inferno is a typical blood flow pattern of benign tumors. We explored the distribution of blood flow of thyroid nodules on color Doppler sonography to provide all possible patterns of blood flow distribution by including weak as well as strong indicators of malignancy. The distribution pattern of blood flow was quantified as: central type, peripheral type, focal thyroid inferno [12], messy type and no blood flow, as shown in Fig. 6.

### Feature weights

It is widely accepted that each feature plays a different role in the identification of benign and malignant thyroid nodules. We proposed the definitions of “benign rate” and “malignant rate” to describe the contribution of each feature based on the statistical results, namely reference weight. Then the final weight of each feature is obtained by the combination of the reference weight and the experience of thyroid experts.

We used the statistical results of ultrasound gray scale features (except cystic features) in literature [9] to obtain the occurrence frequency of each feature on 1658 cases of thyroid nodules. Each feature has a probability of occurrence in both benign and malignant thyroid nodules, as shown in Table 2. The definitions of “benign rate” and “malignant rate” in this paper to show the contribution of each characteristic in the prediction of benign and malignant tumors, respectively, as shown in Eqs. (7, 8):

7 $${\text{BenRate}} = \frac{{N_{iB} }}{{N_{B} }} \div \left( {\frac{{N_{iB} }}{{N_{B} }} + \frac{{N_{iM} }}{{N_{M} }}} \right),\quad N_{B} = 1383,\;N_{M} = 275$$

8 $${\text{MalRate}} = \frac{{N_{iM} }}{{N_{M} }} \div \left( {\frac{{N_{iB} }}{{N_{B} }} + \frac{{N_{iM} }}{{N_{M} }}} \right),\quad N_{B} = 1383,\;N_{M} = 275$$

wherein *N*_*iB*_ (*i *= 1, 2, 3…10) and *N*_*iM*_ (*i *= 1, 2, 3…10) are the numbers of benign and malignant nodules for each grayscale feature, respectively. *N*_*B*_ and *N*_*M*_ represent the total number of benign and malignant nodules, respectively.

**Table 2:** Gray scale ultrasound features of thyroid nodules

Grayscale features	No. of malignant nodules (n = 275)	No. of benign nodules (n = 1383)	Malignant rate	Benign rate
Composition				
Solid	255	805	0.614	0.386
Mixed	20	578	0.148	0.852
Cystic				
Margin				
Ill-defined	68	1262	0.213	0.787
Microlobulated	115	106	0.845	0.155
Shape				
Irregular	92	15	0.968	0.032
Calcification				
Micro-calcification	111	51	0.916	0.084
Macro-calcification	67	179	0.653	0.347
No-calcification	97	1153	0.297	0.703
Taller than wide				
<= 1	134	1326	0.337	0.663
> 1	141	57	0.926	0.074

As for the feature of blood flow, the distribution of blood flow statistics can be obtained by counting the 153 cases of thyroid nodules of Doppler images, as shown in Table 3, followed by the calculation of the benign and malignant rates of blood flow feature of thyroid nodules according to the Formulas (9, 10), as shown in Table 3.

9 $${\text{DBenRate}} = \frac{{N_{iDB} }}{{N_{DB} }} \div \left( {\frac{{N_{iDB} }}{{N_{DB} }} + \frac{{N_{iDM} }}{{N_{DM} }}} \right),\quad N_{DB} = 78,\;N_{DM} = 75$$

10 $${\text{DMalRate}} = \frac{{N_{iDM} }}{{N_{DM} }} \div \left( {\frac{{N_{iDB} }}{{N_{DB} }} + \frac{{N_{iDM} }}{{N_{DM} }}} \right),\quad N_{DB} = 78,\;N_{DM} = 75$$

where *N*_*iDB*_ (*i* = 1, 2, 3…5) and *N*_*iDM*_ (*i* = 1, 2, 3…5) are the numbers of benign and malignant nodules for each distribution type of blood flow, respectively. *N*_*DB*_ and *N*_*DM*_ are the total numbers of benign and malignant nodules for each distribution type of blood flow, respectively.

**Table 3:** Results of blood flow feature statistics of thyroid nodules

	Messy	Central	Peripheral	Focal	No blood flow
No. of malignant nodules (n = 75)	45	6	13	0	11
No. of benign nodules (n = 78)	26	0	20	2	30
Malignant rate (%)	64.3	100	40.3	0	27.6
Benign rate (%)	35.7	0	59.7	100	72.4

The reference weight *W*_*i*_ was obtained according to the benign rate and malignant rate of each feature in Tables 3 and 4, as shown in the Formula (11). The final weight of each feature, malignant score, derived from the opinions of clinical expert and the reference weight, as shown in Table 4.

11 $$W_{i} = \left\{ {\begin{array}{l} {\frac{{R_{iM} }}{{R_{iB} }},\;R_{iB} < R_{iM} \;} \\ {6,\;\left( {{{R_{iM} } \mathord{\left/ {\vphantom {{R_{iM} } {R_{iB} }}} \right. \kern-0pt} {R_{iB} }}} \right) > 6,\quad \left( {i = 1,\;2,\;3 \ldots 15} \right)} \\ {\frac{{R_{iM} }}{{R_{iB} }},\;R_{iB} > R_{iM} } \\ \end{array} } \right. \,$$

wherein *R*_*iB*_ and *R*_*iM*_ is the benign and malignant rate of each feature in Tables 2 and 3, respectively.

**Table 4:** Feature weight of TIRADS

Feature	Weights	
	Feature reference weight (*W*_*i*_)	Malignant weight (score)
Composition		
Solid	1.6	0
Mixed	0.2	0
Cystic		− 2
Margins		
Ill-defined margins	3.7	3
Microlobulated	5.5	6
Shape		
Irregular	6	6
Calcification		
Micro-calcification	6	6
Macro-calcification	1.9	2
No-calcification	0.4	1
Taller than wide		
<= 1	0.5	1
> 1	6	6
Blood flow		
Messy	1.8	1
Central	6	2
Peripheral	0.7	0.5
Focal	0	− 2
No blood flow	0.4	0

### Feature scoring

The malignant risk assessment of thyroid nodules is generally based on the number of malignant features in the current TIRADS [6, 20], which depends on the subjective diagnosis of doctors and does not contain the essential feature of blood flow, so the accuracy is not high. We used different scoring methods for different ultrasound features.

First, the feature scores of composition, calcification and distribution of blood flow were obtained their corresponding malignant weights in TIRADS, as shown in the last column of Table 4.

**Fig. 7:**
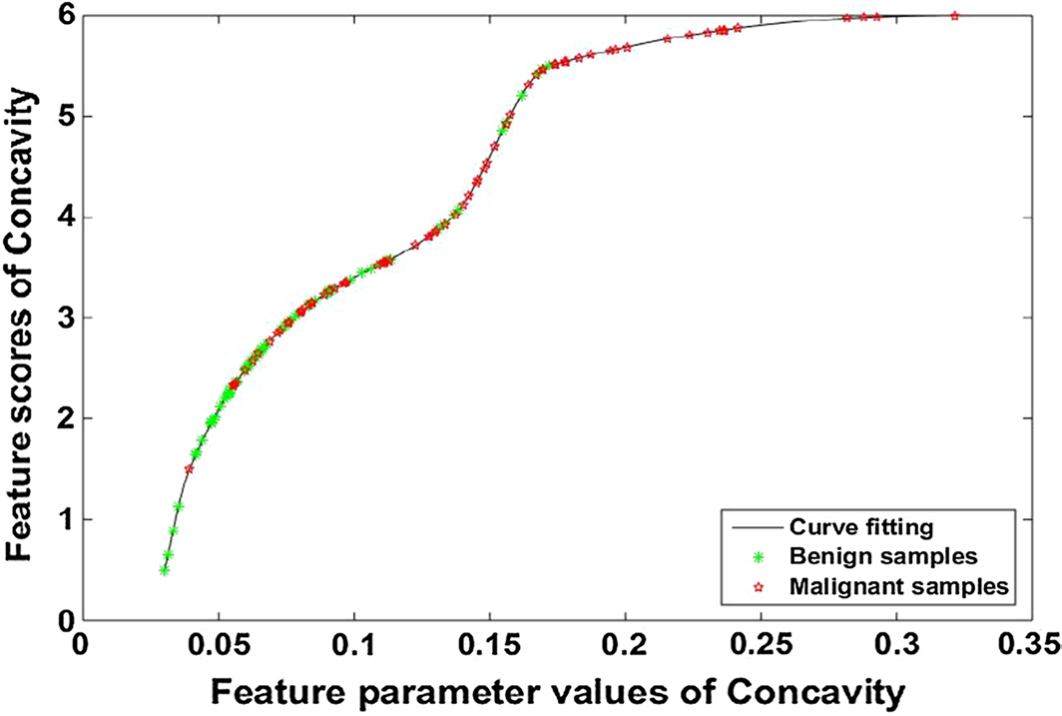
Score curve for the shape feature parameter values of *Concavity*. The abscissa represents the quantified shape parameter value of *Concavity* for each thyroid nodule, and the ordinate represents the feature score for each parameter value. Red “*” represents a sample of malignant thyroid nodules, and green “*” is a benign sample

Then, the feature scores of margin and shape were obtained by the curve fitting of the quantified feature parameters as the abscissa range and their malignant weights as the ordinate maximum. Take the feature score of the parameter Concavity of the shape feature as an example, parameter Concavity of each thyroid nodule were sorted in ascending order as the values of the abscissa. In order to obtain the corresponding feature score for each parameter. We first got the value of maximum, minimum and average of parameter Concavity as x coordinate values, respectively, which corresponded to the feature scores of 6 (the weight of the shape, which is the maximum value of the feature score), 0.5, and 3 as y coordinate values, respectively. Then, the curve fitting was performed on the sample parameters Concavity by these three sets of coordinates so that we can obtain the ordinate values i.e. the feature scores, corresponding to each value of parameters of the abscissa, as shown in Fig. 7. Wherein the red “*” and the green “*” represent the malignant and benign, respectively.

Finally, the feature score of taller than wide (*ScoreAR*) was calculated by the combination of Aspect Ratio parameters and their malignant weights. The feature score was refined in the case of the value of Aspect Ratio greater than 1 combined with the feature weight, instead of being simply divided into two categories in our study to obtain a more accurate malignant assessment, as shown in Formula (12).

12 $$ScoreAR = \left\{ {\begin{array}{*{20}ll} {AR, \quad AR < = 1} \\ {(AR - 1)*w, \quad AR > 1} \\ \end{array} } \right.$$

wherein *AR* is the value of aspect ratio parameter, and *w* is the characteristic weight of taller than wide, *w *= 6, which is shown in Table 4.

### TIRADS score

After accumulating the six category feature—(for a total of eight feature scores, as shown in Table 6 of each thyroid tumor, a comprehensive score of TIRADS for predicting the malignancy of each nodule was obtained, as shown in Formula (13), wherein s_i_ is the score of each feature.

13 $$Score = \sum\limits_{i = 1}^{7} {s_{i} } ,\quad i = 1,\;2,\;3 \ldots ,8$$

**Fig. 8:**
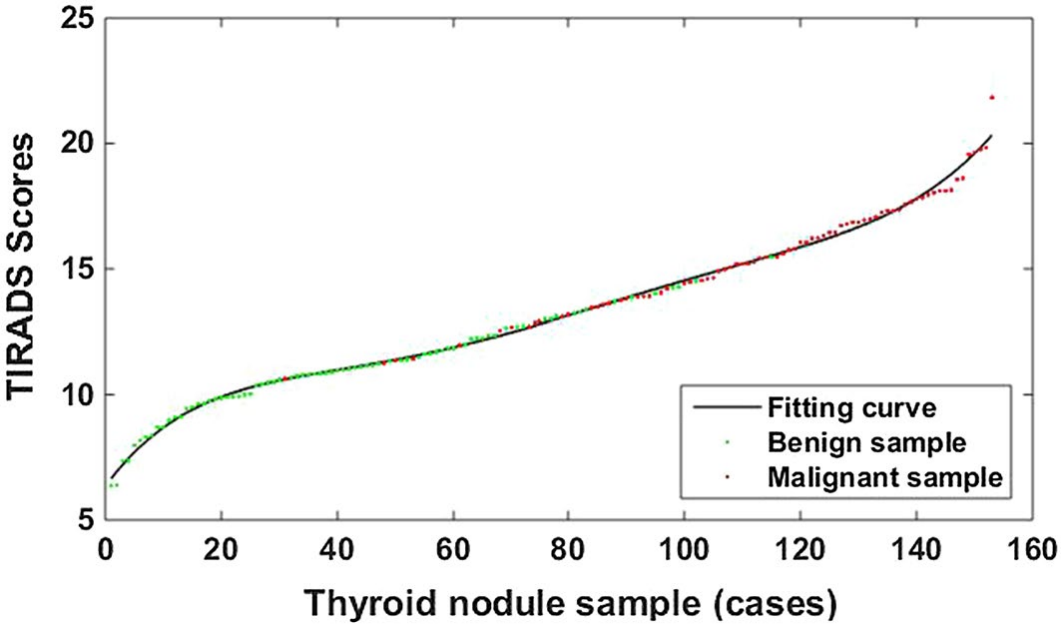
TIRADS score curve of 153 cases of thyroid nodules. The TIRASD score distribution for each thyroid nodule image. Wherein, the red dot represents a malignant sample and green dot is a benign sample, and the black solid line is the fitted curve according to the sample’s TIRADS score

Then, we sorted the TIRADS scores of the 153 cases of the ultra sound image of thyroid nodules in ascending order. The corresponding number of each nodule was taken as the abscissa, and the TIRADS score corresponding to each nodule was used as the ordinate to draw the TIRADS score curve, as shown in Fig. 8

The maximum score of the TIRADS proposed in this paper is 26 points (the sum of the largest malignant weights for each type of feature score), and it is divided into 52 scoring sub-intervals with a 0.5-point step size. First, The TIRADS score for each thyroid nodule was obtained by Eq. 13, and we sorted the TIRADS scores of the 153 cases of the ultra sound image of thyroid nodules in ascending order; then, counted the cases of benign and malignant thyroid nodules whose TIRADS scores fall within each scoring sub-interval. Based on malignant risk of the current TIRADS, these 52 scoring sub-intervals were divided tinto 6 grading intervals, which represent 2, 3, 4a, 4b, 4c, 5 levels in the TIRADS classification. Finally, the number of benign and malignant tumors was counted, and the risk of malignancy was calculated in each TIRADS level according to the Eq. (14).

14 $$R_{i} = \frac{{n_{iM} }}{{n_{iM} + n_{iB} }},\quad i = 1,\;2,\;3 \ldots 6$$

wherein i is the number of TIRADS classification interval, *n*_*iB*_ and *n*_*iM*_ are the numbers of benign and malignant cases in each interval, respectively.

Therefore, the method of TIRADS classification proposed in this paper can obtain the corresponding TIRADS grading and risk of malignant based on the TIRADS score of the thyroid nodule ultrasound image.

## Results and discussion

The experimental data come from the Department of Ultrasound in Beijing China-Japan Friendship Hospital, which do not involve the patient’s personal information.

### Results

**Fig. 9:**
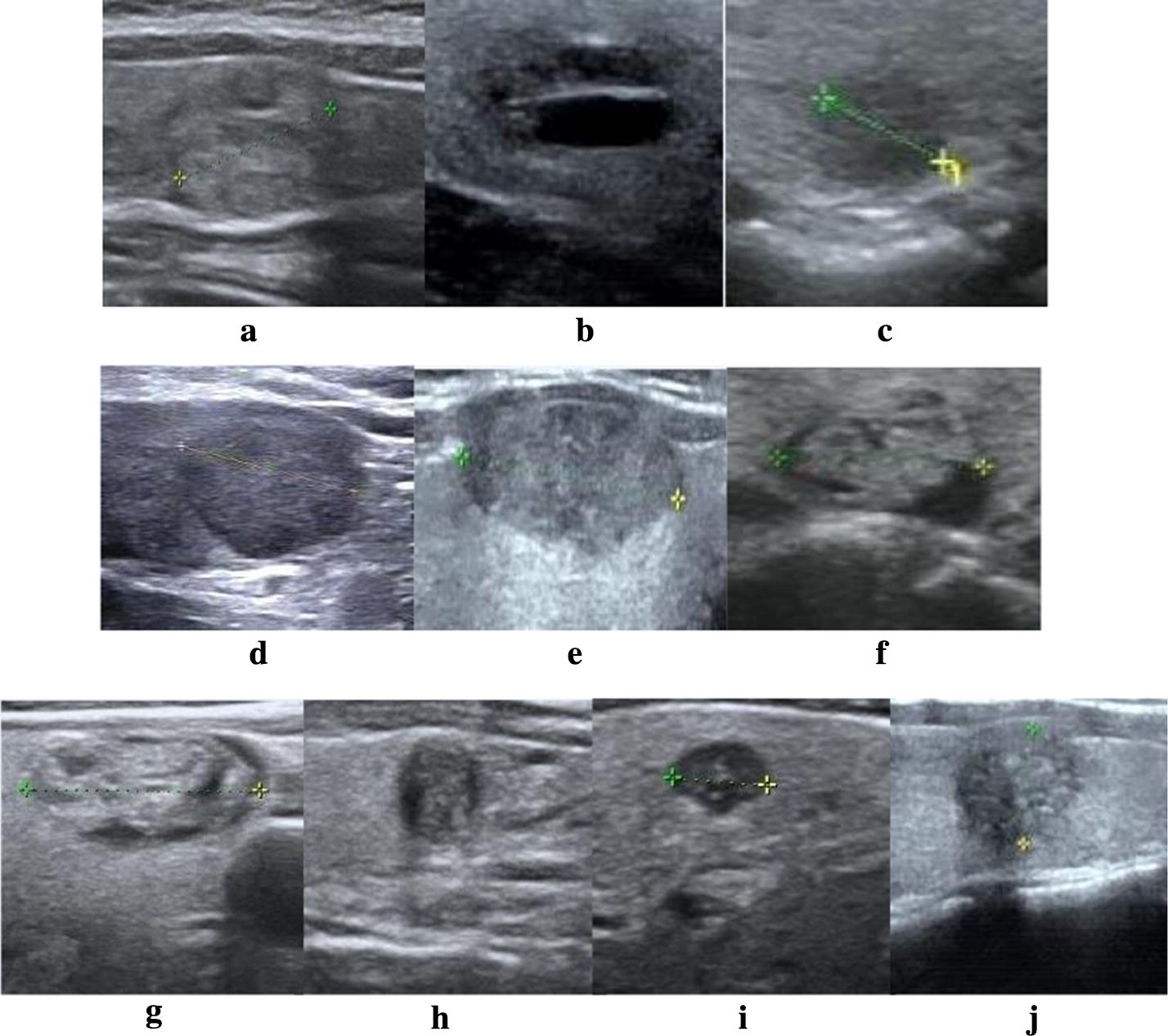
Samples of correct classification results for Ultrasound images of thyroid nodules. Each image is labeled with the TIRADS classification in Table 6

In this study, 153 cases of thyroid nodules were graded, among which 78 were benign and 75 are malignant. The results of the TIRADS classification are shown in Table 5. Samples of correct results for the TIRADS classification are shown in Fig. 9 and their specific parameters of the classification are shown in Table 6.

**Table 5:** TIRADS score and grading results

Range	Total score (score)	No. of malignant nodules (n = 75)	No. of benign nodules (n = 78)	No. of intervals			Cancer risk (%)	TI-RADS
				Malignant	Benign	Total		
1–19	< 9.5	0	0	0	15	15	0	2
20	9.5–10	0	10	1	27	28	3.6	3
21	10–10.5	0	4					
22	10.5–11	1	13					
23	11–11.5	3	8	4	20	24	17	4a
24	11.5–12	1	7					
25	12–12.5	0	5					
26	12.5–13	5	5	9	9	18	50	4b
27	13–13.5	4	4					
28	13.5–14	8	3	17	6	23	74	4c
29–30	14–15	9	3					
31–34	15–17	24	1	44	1	45	98	5
35–52	17–26	20	0

**Table 6:** The specific parameters of the TIRADS classification for each nodule in Fig. 9

Thyroid nodules	Feature parameters								TIRADS		Gender
	Composition	Shape		Margin		Taller than wide	Calcification	Vascularity	Our TIRADS	Radiologist	
		Concavity	Compactness	InterVar	MeanSep						
(a)	Solid	0.035	1.515	8.100	0.109	0.720	No	No	2	2	Benign
(b)	Cystic	0.113	1.478	275.5	2.985	0.623	No	No	3	3	Benign
(c)	solid	0.069	1.441	290.1	3.762	0.965	No	No	4a	4a	Malignant
(d)	solid	0.065	1.476	235.2	2.814	1.069	No	Peripheral	4a	4a	Benign
(e)	Solid	0.072	1.688	257.1	2.480	0.564	No	No	4b	4b	Malignant
(f)	Mixed	0.167	1.577	97.84	1.067	0.742	No	Messy	4b	4b	Benign
(g)	Mixed	0.053	1.710	170.9	1.688	0.516	Macro	Messy	4c	4c	Benign
(h)	Mixed	0.055	1.432	228.2	2.445	1.33	No	Central	4c	4c	Malignant
(i)	Solid	0.111	1.602	657.9	6.143	0.727	Micro	Peripheral	5	5	Malignant
(j)	Solid	0.170	1.668	198.1	2.203	1.155	Micro	Messy	5	5	Malignant

### Discussion

The classification of the thyroid nodules, based onTIRADS in literature [9], is presented in Table 7.

**Table 7:** Classification results of TIRADS in literature [9]

TIRADS category	Benign	Malignant	Total	Risk of malignancy (%)
2	15	0	15	0
3	24	3	27	11.11
4a	22	5	27	18.52
4b	9	10	19	52.63
4c	6	24	30	80
5	2	33	35	94.28

TI-RADS classification of the thyroid is derived from the BI-RADS classification of the breast. BI-RADS of the 2013 edition of the ACR is classified into categories 0–6, with incomplete evaluation of category 0; category 1 negative; category 2 benign; 3 may be benign; 4 suspicious malignant, 4 is divided into three subtypes 4a, 4b and 4c; 5 highly suspected malignant; 6 is pathologically confirmed malignant lesions. Most of the authors have used this method to classify breast and thyroid lesions in the past 3 years, the results of malignant risk comparison are shown in Table 8.

**Table 8:** TIRADS malignant risk comparison results

	Malignancy risk			
	2 (%)	3 (%)	4 (%)	5 (%)
BI-RADS	0	< 5	5–85	> 85
TIRADS in literature 9 [3]	0	11.11	18.5–80	94.28
Our TIRADS	0	3.6	17–74	98

As can be seen from Table 8, the TIRADS presented in this paper is more in line with the Malignancy risk of BI-RADS compared with the Kwak in literature [9].

**Fig. 10:**
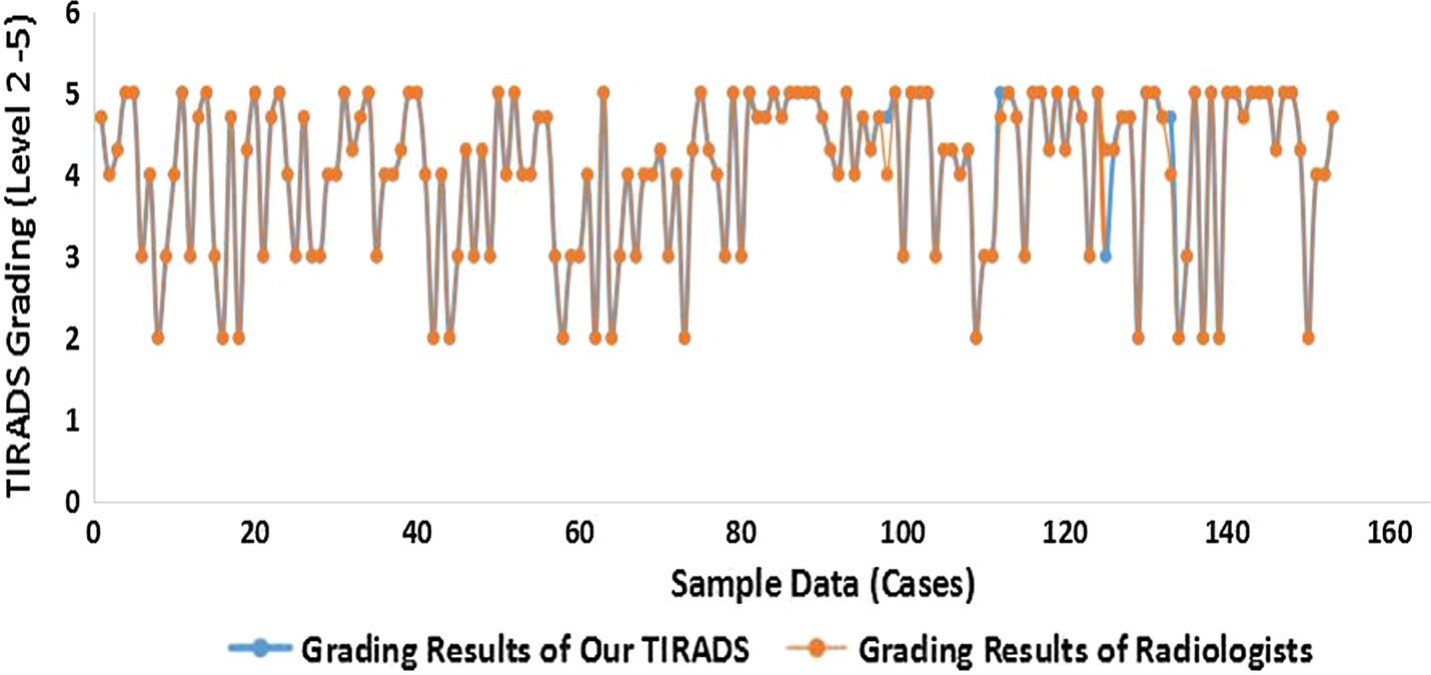
Comparison of TIRADS grading results Comparison of TIRADS grading results between our TIRADS and Radiologists. In order to further confirm the classification accuracy of each sample studied, we compared it with the reference grade of each sample screened by experts, as shown in figure. The abscissa represents the thyroid nodule sample, the ordinate represents the TIRADS grading result corresponding to each sample, the blue represents the TIRADS grading results of this article, and the orange represents the thyroid radiologists grading results

**Fig. 11:**
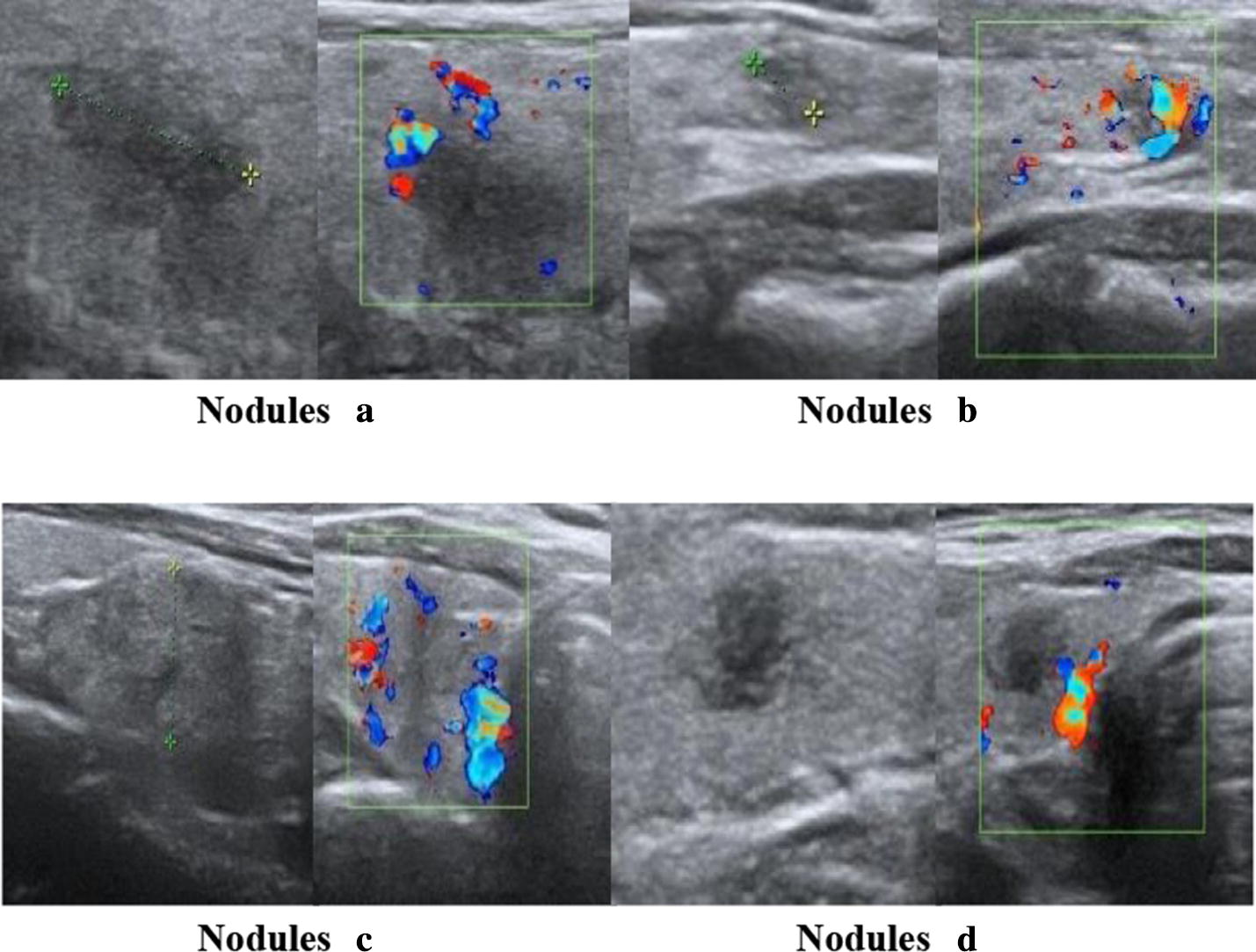
Examples of wrong classification in our TIRADS. Four thyroid nodule samples which classification results are inconsistent with radiologists, named nodules (**a**–**d**). Each sample includes a grayscale ultrasound image and corresponding Doppler color ultrasound image

In order to further confirm the classification accuracy of each sample studied, we compared it with the reference grade of each sample screened by experts, as shown in Fig. 10. In 78 cases of benign nodules, 4 cases of grading results do not match the experts, as shown in Fig. 11, the correct classification rate reaches 94.87% and the classification of malignant nodules is 100%.

In order to find out the reasons for the grading deviation, the TIRADS feature parameters and their TIRADS scores of the each nodules above were analyzed as shown in Table 9.

**Table 9:** The specific parameters of the TIRADS classification in Fig. 11

Features	Weight	Nodules (a)		Nodules (b)		Nodules (c)		Nodules (d)	
		Parameter	Score	Parameter	Score	Parameter	Score	Parameter	Score
Composition	2	Solid	0	Solid	0	Solid	0	Solid	0
Shape									
Concavity	6	0.155	4.557	0.098	3.396	0.047	1.995	0.169	4.962
Compactness	6	1.813	4.479	1.458	2.079	1.654	3.583	1.664	3.680
Margins									
InterVar	3	44.74	1.301	95.38	1.403	14.82	1.129	162.5	1.994
MeanSep	3	0.503	1.301	1.105	1.413	0.194	1.152	1.782	1.972
Taller than wide	4	0.614	0.614	0.667	0.667	0.653	0.653	0.678	0.678
Calcification	6	No	1	Micro	6	No	1	No	1
Vascularity	2	Messy	1	Peripheral	0.5	Peripheral	0.5	No	0
Total Score	30	14.250		15.459		10.015		14.288	
TIRADS	2–5	4c		5		3		4c	
Radiologist	2–5	4a		4c		4a		4a	

The TIRADS classification of nodule (a) is 4c and the reference level of Radiologist is 4a. The thyroid tumor has ill-defined margin, irregular shapes, as can be seen from Table 9. Thus, the malignant scores of shape parameters (*Concavity* and *Compactness*) and margin parameters (*InterVar* and *MeanSep*) are higher according to the weights of margin and shape. Besides, the feature of blood flow in our TIRADS is the messy and experts think that there is no blood flow signals in nodule. Since feature extraction algorithm of blood flow relies on the position of nodule boundary, it is inaccurate boundary information caused by blood flow coverage, which makes the TIRADS score slightly higher than the actual value.

The TIRADS classification of nodule (b) is 5 and the reference grade of radiologist is 4a. It has strong malignant features of ill-defined margin, irregular and micro-calcification, although the tumor is benign. The TIRADS classification of nodule (c) is 3 and the reference grade of radiologist is 4a. It can be seen from Table 9 that the malignant score of all parameters of the nodule is ordinary, and the features of malignant are not very obvious, leading to a lower TIRADS comprehensive score. The experts mainly considered a feature that was not included in the TIRADS classification of this article. That is when the nodule protrudes or protrudes out of the boundary, the risk of malignancy can be increased. However, the grading result is very close to the reference grading of radiology experts.

As for nodule (d), the TIRADS grade is 4c higher than radiologist reference grade 4a. The features of irregular shape and ill-defined margin are strong malignant indicators, as can be seen in Fig. 11. Therefore, TIRADS classification is higher than the reference classification because the two types of features (shape and margin) have higher malignant scores, as shown in Table 9. For thyroid nodules (b) and (d), the experts mainly consider that their features of composition are mixed, while the nodules are solid in our TIRADS. Therefore, the accuracy of boundary information and feature extraction still needs further improvement.

## Conclusion

In summary, we proposed a novel TIRADS to stratify thyroid nodules according to the probability of a malignancy calculated by a scoring system. Although the usefulness of this category system requires confirmation by a prospective study with a general population, our TIRADS could provide helpful guidelines in deciding the optimal strategies for the management of thyroid nodules.
